# Regulation of sebaceous gland homeostasis and sebum secretion by the ERBB receptor family

**DOI:** 10.1016/j.isci.2026.114834

**Published:** 2026-01-30

**Authors:** Theresa Hommel, Marlon R. Schneider, Maik Dahlhoff

**Affiliations:** 1 Laboratory Animal Medicine Unit, Department of Biological Sciences and Pathobiology, University of Veterinary Medicine Vienna, Veterinärplatz 1, 1210 Vienna, Austria; 2 Institute of Veterinary Physiology, Faculty of Veterinary Medicine, University of Leipzig, Leipzig, Germany

**Keywords:** Biomolecules, Molecular mechanism of gene regulation, Cell biology

## Abstract

ERBB receptors are underappreciated regulators of cellular processes in the sebaceous gland (SG). They control sebocyte proliferation, differentiation, and lipid production - mechanisms that are essential for SG homeostasis, sebum secretion, and skin health. Also, impaired ERBB signaling is associated with diseases such as acne, SG hyperplasia, or skin adverse effects to some cancer therapies. By addressing current findings and unsolved aspects of ERBB signaling in the SG, this review aims to encourage deeper investigation to identify potential therapeutic strategies for common and chronic skin conditions.

## Introduction

Sebaceous glands (SGs) are highly specialized exocrine glands whose structure, secretion, and regulation are precisely tailored to protect and maintain our largest organ, the skin. With the exception of the soles and palms, SGs are distributed across the entire human skin and can reach a density of up to 400–900 glands/cm^2^ in areas such as the face.^1^ SG function and sebum composition vary significantly across species and are adapted to the species-specific properties and needs of the skin.^2^ In most animals, the primary roles of SGs include hydrophobic protection, thermal insulation,^3^ and the delivery of pheromones for communication.^4^ In contrast, in the largely hairless human skin, the emphasis lies on the maintenance of the lipid barrier, microbial defense, and protection against UV radiation.^5^ Human sebum combines with the lipids of epidermal keratinocytes to form an acidic, lubricating layer that protects against pathogenic microorganisms and provides a suitable environment for the natural cutaneous microbiota.^6^ However, despite their primary function in sebum production, SGs are now also recognized as hormonally responsive structures involved in local endocrine signaling^7^^,^^8^ and immune modulation.^8^^,^^9^ As a result, they have a substantial impact on the appearance and aging of the skin,^10^ not only by maintaining moisture, elasticity, and structural integrity. Moreover, emerging evidence indicates that SGs may also participate in the systemic energy metabolism.^11^ For this reason, SGs are subject to strict regulation via various receptor systems and intrinsic pathways. For example, alterations in the neuroendocrine system due to stress, hormonal fluctuations, or disease influence sebum secretion via the CRH or melanocortin receptors expressed in the SGs.^8^ Similarly, the stimulation of the insulin receptor or insulin-like growth factor 1 in response to high glucose intake leads to hyperproliferation and increased lipid synthesis via mTOR signaling, resulting in the upregulation of sterol regulatory element-binding protein1 (SREBP1) and peroxisome proliferator-activated receptor gamma (PPARG).^12^ Recent transcriptome analyses have also identified negative regulators of lipogenesis in the sebaceous glands. For instance, oxidized low-density lipoprotein receptor 1 (OLR1) modulates lipid synthesis via the Wnt/β-catenin signaling pathway,^13^ while a second negative regulator of differentiation and lipid storage, NR4A1, exerts its inhibitory effect by directly binding to GATA2, thereby suppressing PPARG activity.^14^

Alterations of the sebaceous gland have been associated with or are acknowledged causative entities of numerous skin diseases (Table 1). The first group includes diseases in which the sebaceous glands are hypoplastic to atrophic and produce reduced amounts of sebum. According to current knowledge, the changes in the sebaceous glands are not causally involved in the pathogenesis of those conditions but rather represent secondary effects. Nevertheless, the reduction in sebum secretion can contribute to the clinical presentation. Examples include atopic dermatitis (AD), psoriasis, and specific forms of alopecia.^29^ Skin diseases for which the sebaceous gland has a causative role include acne,^15^^,^^29^ seborrhea,^19^ as well as sebaceous gland hyperplasia and carcinoma.^25^ The latter group of diseases will be discussed in detail in the appropriate section (“The ERBB network family in sebaceous gland pathology”).

**Table 1:** Skin diseases with sebaceous gland alterations

Skin disease	Clinical findings	Sebaceous gland alteration/involvement	Secondary factors	Category of skin disease	Reference
Acne	Non-inflammatory lesions (comedones) and inflammatory lesions (papules, pustules, severe cases may develop into cysts)	Increased sebum production, altered sebum composition, formation of comedones, release of proinflammatory cytokines	Colonization with Cutibacterium acnes	SG plays a causative role and ERBB Network Family SG Pathology	Clayton et al.^15^; Choudhury et al.^16^; Williams et al.^17^; Corvec et al.^18^
Seborrhea	Oily skin	Enlarged sebaceous glands, excessive sebum production	NA	SG plays a causative role and ERBB Network Family SG Pathology	Smith and Thiboutot^19^
Seborrheic dermatitis	Patches of mild to severe rash with greasy yellow scales	Increased sebum production	Colonization with Malassezia	SG plays a causative role	Choudhury et al.^16^; Guttman-Yassky et al.^20^
Atopic dermatitis	Eczematous rash, pruritus, xerosis	Sebaceous hypoplasia, resulting in reduced sebum production, impairment of the skin lipid barrier	Altered skin microbiome	SG is not causally involved	Choudhury et al.^16^; Guttman-Yassky et al.^21^
Psoriasis	Well-demarcated erythematous plaques covered with silvery-white scales, akanthosis, pruritus	Atrophy of the sebaceous glands, but no changes in sebum composition or lipid barrier content	NA	SG is not causally involved	Choudhury et al.^16^; Griffiths and Barker^22^
Rosacea	Transient or persistent central facial redness with papules, pustules, and telangiectasia, sometimes also involving the eyes, and long-term phymatous changes	Altered sebum composition, reduced barrier function	NA	SG is not causally involved	Choudhury et al.^16^; van Zuuren et al.^23^
SG hyperplasia	In most cases, multiple 1–5 mm-sized papules with a soft, pale yellow or skin-colored appearance	Reactive hyperproliferation of sebocytes with normal glandular architecture	NA	SG plays a causative role and ERBB Network Family SG Pathology	Weedon^24^
SG adenoma	Skin-colored to yellowish papule or nodule, occasionally ulcerated	Benign, autonomous, disorganized hyperproliferation of sebocytes with basaloid expansion	NA	SG plays a causative role	Weedon^24^; Ferreira et al.^25^
SG carcinoma	Periocular: nodule, sometimes ulcerated Extraocular: single erythematous nodule, sometimes with yellowish-brown areas, can reach sizes of several centimeters, frequently ulcerated	Malignant transformation of sebaceous glands, infiltrative growth of pleomorphic basaloid cells	NA	SG plays a causative role and ERBB Network Family SG Pathology	Ferreira et al.^25^; Alsaad et al.^26^; Park et al.^27^
Cicatricial alopecia	Irreversible hair loss with scarring	Inflammation of sebaceous glands and ducts, atrophy of the sebaceous glands	NA	SG is not causally involved	Al-Zaid et al.^28^

The epidermal growth factor receptor (EGFR/ERBB) system, whose roles in various organ systems have already been intensively discussed due to its increasing importance in cancer therapy, also appears to have a non-negligible effect on SG proliferation and differentiation. This review provides a comprehensive overview of the current knowledge on the importance of the ERBB receptor system for the physiology and pathology of the SGs and the consequences of its dysregulation for cutaneous pathologies. Furthermore, we aim to identify and discuss emerging research directions and key questions that need to be addressed due to the growing importance of ERBB-specific therapeutics in cancer medicine.

## Sebaceous gland homeostasis

The SG is an integral component of the cutaneous adnexal system, yet it remains largely overlooked in translational biomedical research. Typical SGs are morphologically and functionally associated with hair follicles and the arrector pili muscle, forming the pilosebaceous unit.^30^ In addition, there are ectopic sebaceous glands that are not hair follicle-associated, including specialized glands such as the Meibomian glands of the eyelids, which contribute to tear film stability and ocular surface protection through lipid secretion.^31^ SGs secrete sebum, a complex lipid-rich mixture with a characteristic, species-specific composition, via holocrine secretion.^30^ In humans, sebum consists of triacylglycerides, diacylglycerides, FFAs, wax esters, squalene, and cholesterol.^19^ Wax esters and squalene are unique to the SGs and are not found elsewhere in the body, at least in such high concentrations. The holocrine secretion process entails progressive intracellular lipid accumulation, terminal sebocyte differentiation, and eventual cell lysis, resulting in the release of cellular contents into the hair canal and subsequent distribution on the skin surface.

The pilosebaceous unit develops in distinct stages from parts of the surface ectoderm.^32^ Already in the early stages of hair follicle formation, cells expressing the markers SOX9 (SRY-related HMG box gene 9) and LRIG1 can be identified. SOX9-positive cells are essential for the development of the SG, as their deletion prevents SG development in the embryo.^33^ Once a sufficient number of LRIG1-positive cells have formed, they divide into clusters and further differentiate into the respective sebocyte compartments (SEB), comprising the basal proliferation layer, maturing sebocytes, and the excretory duct, to form the typical lobular gland structure. In parallel, a stem cell population of Lrig1-positive cells establishes itself, which is responsible for the continuous maintenance and homeostasis of the SG. These SG stem cells are located directly adjacent to the junctional zone of the hair follicle. The cell population of the junctional zone is very heterogeneous, and the specific subpopulation from which the sebaceous progenitor cells evolve has not yet been identified.^34^ However, previous literature described the expression of GATA-binding factor 6 (GATA6), leucine-rich repeat-containing G-protein-coupled receptor 6 (LGR6), and leucine-rich repeats and immunoglobulin-like domain protein 1 (LRIG1) within this anatomical unit^33^^,^^34^^,^^35^ GATA6-positive populations primarily maintain the duct and infundibulum, while GATA6-negative, LRIG1-positive cell populations subsequently proliferate to maintain the SG. Interestingly, the embryonic stimulation of EGFR leads to SG enlargement and an expanded LRIG1-positive stem cell pool.^36^

**Figure 1:**
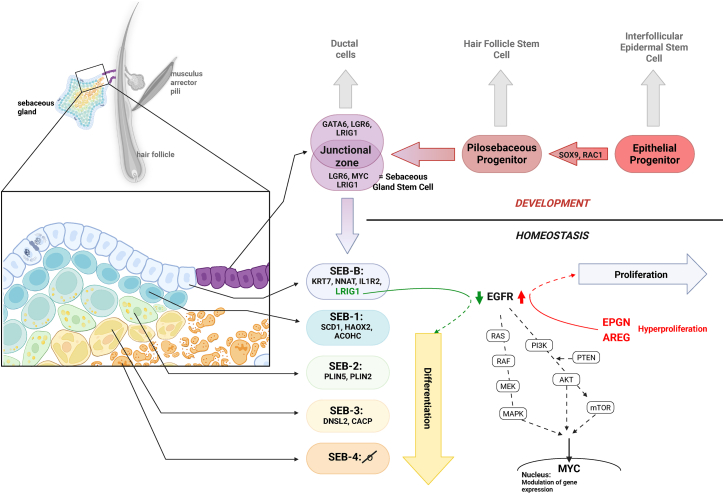
Schematic overview of pathways governing sebaceous gland development and homeostasis, including exemplary markers for the individual stages Sebaceous gland stem cells arise embryonically from epithelial progenitor cells within the pilosebaceous unit and concentrate in the junctional zone. From there, they generate sebocytes (SEB), whose homeostasis in the sebaceous gland appears to be influenced by the ERBB receptor system in interaction with the marker protein LRIG1. Hypothetical connections are depicted as dashed, proven ones as solid lines. Confirmed markers and effector proteins are printed in bold, while hypothetical markers and effector proteins, which are strongly supported by receptor biology, are printed in normal font. Structures of the interfollicular epidermis (IFE) that do not belong to the sebaceous gland itself are shown in gray. Created with BioRender software (BioRender, Toronto, Canada)

During homeostasis, sebocyte maturation follows a centripetal gradient, originating from a peripheral basal layer.^37^ In the adult SG (Figure 1), this most peripheral sebocyte layer (SEB-B) expresses markers such as keratin 7 (KRT7) or interleukin-1 receptor type 2 (IL1R2).^34^ Once these sebocytes progress to the next stage of differentiation (SEB-1), enzymes such as stearoyl-CoA desaturase 1 (SCD1),^33^ hydroxyacid oxidase 2 (HAOX2), and aconitase 1 (ACOHC) become extensively present.^34^ As maturation progresses, SEB-2 cells increase perilipin 2 (PLIN2) and perilipin 5 (PLIN5) levels.^38^ PLIN2 serves as a marker for lipid droplets, which are increasingly stored and enriched with lipids in the course of sebocyte maturation. SEB-3 sebocytes, the last differentiation stage with detectable transcripts, are characterized by expression of carnitine O-acetyltransferase (CACP) and deoxyribonuclease-1-like 2 (DNSL2), with the latter being an important trigger for programmed cell death, which is necessary for holocrine secretion.^34^^,^^39^ During this differentiation process, repeated phases of metabolic reprogramming occur, through which the cells adapt to changing conditions of nutrient and oxygen availability.^40^^,^^41^ Metabolically active sebocytes are programmed for active *de novo* lipogenesis (DNL) from glucose. The central metabolic pathway is therefore glycolysis, which is not only used for ATP production via the TCA cycle, the electron transport chain (ETC), and oxidative phosphorylation (OXPHOS), but also to provide metabolites for biosynthetic processes. In particular, citrate, which is exported from the TCA cycle, is a key substrate for cholesterol and squalene synthesis and production of FAs for the formation of TAGs, wax esters, and phospholipids. In addition to glucose, glutamine, ketone bodies, and exogenous FAs are introduced into the citrate cycle in smaller amounts to support lipid biosynthesis.^40^ The expression of lipogenesis-associated genes is essentially controlled by transcription factors such as sterol regulatory element-binding proteins (SREBPs) and PPARG.^42^ These in turn are subject to regulation via upstream signaling pathways, including PI3K-AKT and mTOR, which respond to metabolic hormones such as insulin and leptin,^12^^,^^43^ but also to growth factor receptors such as ERBBs and inflammation-related receptors such as the Toll-like receptors (TLRs).^44^ The maturation process is accompanied by a loss of proliferative capacity. Here, the downregulation of the expression of the ERBB receptors during sebocyte maturation contributes to inhibiting proliferation and promoting differentiation. A defective downregulation of ERBBs can result in uncontrolled proliferation and malignant transformation. Despite the critical role of SGs in skin homeostasis and barrier function, the molecular mechanisms governing sebocyte lineage commitment, differentiation, and holocrine cell death remain poorly elucidated. Recent findings indicate a pivotal role for the ERBB receptor tyrosine kinase family in the regulation of SG homeostasis, including progenitor cell proliferation and sebocyte differentiation.

### The ERBB receptor family: Basic biology

The EGFR/ERBB family consists of four receptors, EGFR (ERBB1, HER1), ERBB2 (NEU, HER2), ERBB3 (HER3), and ERBB4 (HER4), which can be activated by eleven ligands. After ligand binding, the receptors form homo- or heterodimers, followed by the auto-phosphorylation of cytoplasmic tyrosine residues.^45^ EGFR is activated by at least seven ligands: epidermal growth factor (EGF), transforming growth factor alpha (TGFA), heparin-binding EGF-like growth factor (HBEGF), epiregulin (EREG), amphiregulin (AREG), betacellulin (BTC), and epigen (EPGN). ERBB4 is responsive to BTC, HBEGF, EREG, and the neuregulin family members NRG1–4, whereas ERBB3 is selectively activated by NRG1 and NRG2.

### Epidermal growth factor receptor

EGFR is expressed in a wide range of organs and tissues and plays a crucial role in tissue homeostasis, particularly in processes such as proliferation, differentiation, and migration.^46^ In human skin, EGFR is expressed in all layers of the epidermis, hair follicle, and SG.^47^ Depending on their genetic background, mice lacking EGFR die during midgestational age or survive up to a few weeks after birth. Abnormalities of the skin, lungs, gastrointestinal tract, brain, and bones can be observed in surviving animals.^48^^,^^49^^,^^50^^,^^51^ In tumors, EGFR is frequently mutated at various sites, resulting in its hyperactivation. This leads to the sustained activation of several kinases, such as MAPK (mitogen-activated protein kinase), as well as the activation of RAS (GTPase Ras). EGFR plays a significant role in the pathogenesis of e.g., head and neck cancer,^52^ colorectal cancer,^53^ and non-small cell lung cancer.^54^ EGFR also plays an important role in skin tumors, which will be discussed later in discussion.

### ERBB2

ERBB2 is a ligand-less orphan receptor that needs a dimerization partner (EGFR, ERBB3, or ERBB4) to be activated. ERBB2 is important during embryonic development: ubiquitous ERBB2 knockout is lethal at mid-gestation due to heart and neural defects.^55^^,^^56^ This non-autonomous ERBB receptor is often upregulated in many cancer types, such as breast, lung, stomach, bladder, and ovarian cancer^57^^,^^58^^,^^59^^,^^60^ and is an important target for cancer therapy. ERBB2 is expressed in the epidermis of the skin and in proliferative cells of the pilosebaceous unit.^61^ Skin-specific ERBB2 knockout mice revealed that ERBB2 is dispensable for skin development and homeostasis, but a two-stage chemical skin carcinogenesis experiment found that ERBB2 has an important role in skin tumorigenesis.^62^ Furthermore, this receptor can be activated ligand-independently by UV irradiation^63^ and increases UV-induced skin tumorigenesis.^64^^,^^65^ Transgenic overexpression of ERBB2 in mice causes spontaneous tumor formation.^61^^,^^66^^,^^67^^,^^68^

### ERBB3

ERBB3 is the second non-autonomous receptor of the ERBB family. It can be bound by three ligands, but its intrinsic kinase domain is catalytically inactive. As ERBB2 knockout mice, ERBB3-deficient mice die during mid-gestation due to cardiac and neuronal defects.^69^^,^^70^ ERBB3 is expressed throughout the epidermis with higher levels in the suprabasal and spinous layers and additional expression in hair follicles.^61^ ERBB3 has also been detected in cutaneous tumors^71^^,^^72^ and plays an important part in murine induced skin carcinogenesis.^73^ As ERBB3 is the only ERBB family member that lacks catalytic kinase activity,^74^ its potential functions in cancer biology have been ignored for a long time. Today, there is evidence that ERBB3 is also overexpressed in various tumor types, including breast, ovarian, prostate, colon, and lung cancer, correlating with a poor clinical prognosis.^75^ In fact, the ERBB2/3 heterodimer is the most active signaling dimer in the ERBB family^76^ and is often hyperactive in tumors, making ERBB3 an important oncogenic receptor.

### ERBB4

ERBB4, such as EGFR, is again an autonomous receptor and essential for embryonic development, as are ERBB2 and ERBB3. Mice lacking ERBB4 die during mid-gestation due to cardiac and neuronal defects.^77^ In the epidermis, ERBB4 is expressed in the basal layer, and its deletion in keratin 5 (KRT5) expressing cells significantly reduces their proliferation.^78^

## ERBB receptor family in sebaceous glands

Although the EGFR is named after its effect on epidermal cells and it plays essential roles in keratinocyte proliferation, differentiation, and cutaneous repair processes, all four ERBB receptors are widely expressed across the body. Moreover, all ERBB receptors are present in the SG, with EGFR showing the strongest expression. Since no SG-specific driver mouse line targeting the whole gland is currently available,^79^ *in vivo* studies of the ERBB receptor system in SGs rely on KRT5-Cre and KRT14-Cre driver mice. In these mice, ERBB receptors are deleted both in the epidermis and in the pilosebaceous unit. A number of these mouse models for ERBB receptors and ERBB ligands still exhibit strong, specific phenotypes in the SGs (summarized in Table 2), and will therefore be discussed in this context.

**Table 2:** Summary of SG phenotype of genetically modified mouse models

Receptor/Ligand	Mutation	Phenotype	Reference
EGFR	*Egfr* Leu863Gln (Dsk5)	Hyperplasia of SG and increased sebum production due to increased EGFR activity	Dahlhoff et al.^80^
ERBB2/3	KO (*Krt5-Cre*)	Hyperplasia of SG due to the loss of ERBB2 and ERBB3	Hommel et al.^81^
ERBB2	TG (*Krt5-Erbb2*)	Hyperplasia of SG due to increased ERBB2 activity	Kiguchi et al.^61^
NRG3	TG (*Krt14-Nrg3*)	Hyperplasia of SG due to a highly active ERBB4 receptor	Panchal et al.^82^
TGFA	TG (*Mmtv-Tgfa*)	SG hyperplasia due to the overexpression of TGFA	Halter et al.^83^
AREG	TG (*Krt5-Areg*)	Hyperplasia of SG due to the overexpression of AREG	Li et al.^84^
EPGN	TG (*Cba-Epgn*)	Hyperplasia of SG; increased sebum production due to ubiquitous EPGN overexpression	Dahlhoff et al.^85^
EPGN	TG (*Krt5-Epgn*)	Hyperplasia of SG; increased sebum production	Dahlhoff et al.^36^

TG: transgenic, KO: knockout, *Krt5*: keratin 5 promoter, *Krt14*: keratin 14 promoter, *Cba*: chicken β-actin promoter, *Mt*: metallothionein promoter, Leu: leucine, Gln: glutamine.

EGFR/ERBB receptors appear to be important for the proliferation of (peripheral) SG progenitor cells, while the receptors are downregulated as sebocyte differentiation proceeds.^86^ This fits well with a more general model, also applicable to other tissues, which ascribes to these receptors pivotal functions in the rapid clonal expansion of progenitors (transient amplifying cells) to generate an appropriate number of differentiated cells.^87^

### Epidermal growth factor receptor

All seven EGFR ligands are expressed in the SG, with AREG, HBEGF, and EPGN being more highly expressed during differentiation, while BTC is downregulated.^86^ Dsk5 mice, which have a hyperactive EGFR due to a point mutation, show increased proliferation and hyperplasia of the SG, along with significantly increased sebum production and a thicker sebum layer on the coat.^80^ Transgenic mice that overexpress EPGN or AREG during embryonic development also show SG hyperplasia and pronounced seborrhea, mediated through EGFR activation.^36^^,^^84^^,^^85^ Overexpression of TGFA with mouse mammary tumor virus (MMTV) resulted in SG hyperplasia but not seborrhea.^83^ These results highlight the importance of the EGFR and its ligands for the development and homeostasis of the SG. The activation of EGFR induces a downstream signaling cascade via PI3K-AKT, RAS-RAF-MEK-ERK, and phospholipase C (PLCG) signaling pathways.^46^ These cascades lead to the induction of various transcription factors. In sebaceous glands, the myc proto-oncogene protein (MYC) plays a central role, as it regulates the transition of cells from the stem cell compartment to actively proliferating and differentiating cells.^88^ Accordingly, MYC was found to be significantly increased in studies on Dsk5 mice and by the overexpression of EPGN.^36^^,^^80^^,^^85^

Another important regulator of early sebocyte development and stem cell marker for sebocyte progenitor cells is LRIG1.^89^ LRIG1 is a negative feedback regulator of EGFR and other ERBB receptors and modulates cell proliferation by acting as a negative feedback regulator of the ERBBs.^90^^,^^91^ Ubiquitous LRIG1 knockout mice showed a psoriatic phenotype comparable to that of mice with increased ERBB receptor activity stimulated by the transgenic expression of the ligand AREG in the skin and sebocytes.^92^^,^^93^ During sebocyte differentiation, LRIG1 is degraded, and the ERBB receptors are stabilized on the cell surface of sebocytes, leading to a switch from proliferation to differentiation. In contrast, if EGFR is inhibited by monoclonal antibodies (MABs) during cancer therapy, such as with Cetuximab, patients often develop seborrhea with hyperplastic SGs.^94^ Skin-specific overexpression of LRIG1 also resulted in SG hyperplasia due to increased proliferation.^95^ However, unlike the knockouts, LRIG1-overexpressing mice develop impaired skin homeostasis, which was demonstrated by the expression of keratin 6 in the epidermis. The overexpression of LRIG1 leads to the increased activation of NOTCH1 and its downstream targets, recombining binding protein suppressor of hairless (RBPJ), mastermind-like protein 1 (MAML1), MYC, and transcription factor HES-1 (HES1), which causes alopecia, hyperproliferation of the sebaceous glands, and a thickened epidermis.^95^ This is likely to explain the apparently contradictory phenotypes of LRIG1 knockout, LRIG1 overexpression, and EGFR hyperactivation models.

### ERBB2/3

For ERBB2, not the skin-specific knockout^62^ but rather an overexpression of ERBB2 results in pronounced general hyperproliferation with the enlargement of the sebaceous glands, as shown in ERBB2 transgenic mice overexpressing wild-type rat ERBB2 in the epidermis and sebaceous glands.^61^ But interestingly, SG hyperplasia also occurs as a result of simultaneous skin-specific deletion of ERBB2 and ERBB3,^81^ while publications in which either ERBB2^62^ or ERBB3^73^ were deleted alone in the skin did not report enlarged SGs in the skin of the knockout mice. Therefore, it is likely that one receptor is sufficient to compensate for the missing one. Such compensation mechanisms are supported by data from SZ95 sebocytes, in which a knockdown of ERBB3 leads to a significant upregulation of ERBB2 expression.^86^ It was demonstrated in HaCaT cells that in the skin, especially ERBB1/2 or ERBB1/3 heterodimers play an important role, since EGFR phosphorylation remains largely unaffected after a single knockout of either ERBB2 or ERBB3, whereas the simultaneous deletion of both receptors causes a significant reduction of EGFR phosphorylation due to the inability to form functional heterodimers.^81^ These findings highlight the critical role of ERBB2 and ERBB3 heterodimers in maintaining EGFR signaling and driving skin and sebocyte differentiation, thus providing an explanation for the fact that skin-specific ERBB2/ERBB3 double-knockout mice resemble the phenotype of EGFR inhibition. This mouse model further reflects potential adverse effects of cancer therapy with MABs or bispecific ABs targeting the ERBB2 and ERBB3 receptors.

### ERBB4

The activation of the ERBB4 receptor by NRG3 leads to SG hyperplasia,^82^ analogous to EGFR hyperactivation, and can also be attributed to the increased proliferation of SG sebocytes. However, the deletion of the ERBB4 receptor does not impair SG development or homeostasis.^78^

## The role of ERBBS in lipogenesis

The secretion of sebum relies on the correct differentiation and lipogenesis of sebocytes and requires precise regulation of the underlying metabolic processes. Although the involvement of ERBB receptors in the synthesis of lipids in the SG has only been investigated to a limited extent, initial studies already indicate that sebocyte lipid metabolism is influenced by ERBB signaling. Clinical studies showed a reduction of squalene and TGs during the targeted treatment of the EGFR^96^ and the individual knockdown of EGFR, ERBB2, and ERBB3 in SZ95 sebocytes directly affected the composition of lipids. Here, the downregulation of EGFR and ERBB3 increased the abundance of TGs, primarily with more than 48 carbon atoms, and the downregulation of all receptors studied increased the presence of cholesterol esters but not cholesterol. This knockdown was accompanied by an increase in the transcription of proteins involved in fatty acid synthesis, such as fatty acid synthase (FAS) via the SREBPs as transcription factors.^86^ In contrast, studies on the effects of ERBB receptor activation in sebocytes did not provide a consistent picture. They appear to be sensitive to the cell environment. Using the SZ95 sebocyte cell line, it was shown that EGF stimulation leads to either positive or negative regulation of lipid metabolism and differentiation depending on the presence of palmitic acid.^97^

A respective ERBB signaling axis via PI3K-AKT-mTORC1 has already been described in other physiological as well as pathological conditions and appears to be a central mechanism for lipid synthesis. In tumors, it promotes *de novo* lipogenesis (DNL) both via the direct activation of ATP citrate lyase (ACLY) and via the transcriptional control of lipogenic genes through the mTORC1-dependent activation of SREBP1.^98^ In colon carcinoma cells, for example, the activation of EGFR via its ligand EGF led to increased lipid droplet density.^99^ Similarly, ERBB2 is involved in the regulation of DNL, resulting in increased FA synthesis in tumor tissue in ERBB2-positive breast cancer. Therefore, targeted therapy of the ERBB2 receptor with MABs leads to an impairment of DNL due to reduced FASN expression in cancer cells despite high glycolysis. Cancer cells attempt to compensate for this by increasing FA uptake, which is already being discussed as a potential new therapeutic target.^100^ ERBB4 has been shown to regulate cholesterol biosynthesis and LDL uptake by activating SREBP-2 after binding its ligand NRG1. Therefore, a pan-ERBB inhibitor such as lapatinib was able to suppress NRG1-induced cholesterol biosynthesis, in contrast to an EGFR-specific inhibitor such as erlotinib.^101^ There is also evidence of the involvement of ERBBs and their ligands in lipid storage in white adipose tissue. Initial patient data and *in vitro* studies suggest that the EGFR, ERBB2, and ERBB4 regulate lipogenic genes such as *FASN* in pre-adipocytes and fully differentiated cells.^102^

Even though these molecular mechanisms have so far not been systematically investigated in the SG, the results from other tissues suggest that comparable ERBB-mediated signaling pathways could also contribute to the regulation of lipogenesis with potential implications for dermatological diseases such as acne or seborrhea or side effects of ERBB cancer therapy, which is why a focus on this aspect could be worth considering in the future.

## The ERBB network family in sebaceous gland pathology

The dysregulation of ERBB signaling is associated with various skin pathologies, including inflammatory dermatoses such as psoriasis and impaired wound healing, which are often associated with abnormal proliferation or differentiation defects.^103^^,^^104^ Furthermore, aberrant ERBB receptor activation contributes to the pathogenesis of various skin cancers, including squamous cell carcinoma (SCC), basal cell carcinoma, malignant melanoma, and SG carcinoma.^105^^,^^106^ Notably, overexpression of EGFR ligands such as EGF, HBEGF, and TGFA is commonly observed in cutaneous malignancies, further underscoring the oncogenic potential of the ERBB axis in skin biology. Mutations in EGFR have been found in approximately 7% of patients with cSCC and are considered a poor prognostic factor in this context.^107^ In melanoma, however, EGFR mutations are rare; rather, EGFR overexpression or activation of downstream signaling pathways has been associated with tumor progression and resistance to targeted therapies. In SG carcinoma, EGFR and ERBB2 are strongly expressed, and both enhance the proliferation of the transformed sebocytes.^108^^,^^109^ Studies showed that EGFR, ERBB2, and ERBB3 are as important for proliferation and tumor initiation in sebaceous gland carcinoma as they are in keratinocytes of cutaneous squamous cell carcinoma.^62^^,^^73^^,^^110^

Sebaceous gland hyperplasia is a benign enlargement of the SG characterized by strongly proliferating sebocytes. In experimental models, hyperplasia of the SG often results from the overexpression of growth factors. The EGFR can stimulate the proliferation of LRIG1-positive SG progenitor cells via the transcription factor MYC and the overexpression of EGFR ligands such as epigen.^36^^,^^85^ As noted before, AREG,^84^ TGFA,^111^ and EGF^112^^,^^113^ lead to SG hyperplasia, and similar effects are elicited by the expression of hyperactive EGFR, such as in Dsk5 mice.^80^ These studies demonstrate that EGFR plays a critical role in the proliferation of SG progenitor cells and may therefore be essential for SG development and for SG hyperplasia.

However, there is also growing evidence that the skin microenvironment has a substantial influence on the development and progression of cutaneous malignancies such as SG carcinoma, SCC, and malignant melanoma. Since the sebaceous glands contribute essentially to the composition of the lipid envelope of the skin, changes in sebum composition due to a disruption in the regulation of ERBB receptors might impair this sensitive microenvironment. Pharmacological intervention of EGFR, for example, leads to a change in the proportion of squalene in sebum.^96^ This is relevant as oxidized squalene, known as squalene peroxides, can have potentially carcinogenic effects when exposed to UV radiation and contribute to the development of cutaneous SCC.^9^ It has also been observed that different environmental lipid compositions can influence not only proliferation but also metastatic behavior and organ tropism in melanoma cell lines. Specific components such as ceramides, whose secretion by the sebaceous gland is influenced by ERBB2 and ERBB3,^81^ promote IL6 secretion and favor the liver tropism of melanoma cells, while phosphatidylcholine, on the other hand, was correlated with increased lung metastasis.^114^ Although the exact role of ERBB receptors in this context has not yet been sufficiently investigated, these observations underline the importance of the cutaneous microenvironment as a potential modulating factor in skin carcinogenesis and require further research.

Acne vulgaris (acne) is a skin disorder in humans that primarily occurs during puberty and most intensively affects the face and torso. Characteristics of acne vulgaris are increased sebum production, altered sebum composition, and the formation of comedones in the infundibulum of the hair follicle. The most common causes of acne are hormonal changes in androgens during puberty and growth factors, but the ERBB receptors may also play a role in acne, as they frequently regulate the growth and differentiation of epithelial cells.

Inhibition of EGFR through Cetuximab or other monoclonal antibodies (MABs) or tyrosine kinase inhibitors (TKIs) very frequently leads to acne, xerosis, pruritus, skin pain, and rash in patients with cancer.^94^^,^^115^ A study of patients treated with cetuximab has demonstrated that these symptoms are associated with a change in sebum composition during therapy, with a reduction in the proportion of squalene and triglycerides.^96^ In line, in cell culture, it has been shown that knockdown of EGFR in mature sebocytes,^116^ such as the SZ95 cell line, leads to increased lipid production and an altered lipid composition.^86^ Treatment with EGF results in reduced lipid production and upregulation of inflammatory genes in isolated sebocytes and in patients with acne, likely because EGF stimulates the proliferation of progenitor cells, which negatively impacts sebocyte maturation.^117^

Seborrhea is caused by excessive sebum production from hyperplastic sebaceous glands. The classic phenotype known as oily skin in humans can also be observed as greasy-appearing fur in mouse models with excessive EGFR activation, for example, after the transgenic expression of AREG^84^ or EPGN.^85^ According to current knowledge, seborrheic dermatitis is the result of secondary colonization by Malassezia. These yeasts hydrolyze saturated sebum lipids, producing unsaturated fatty acids that irritate the skin and trigger an inflammatory reaction, leading to the classic appearance of reddish patches and greasy yellow scales.

Rosacea is likewise associated with sebaceous glands. Besides the correlation between the location of reported facial redness and papules and the sebaceous glands, and the perisebaceous infiltration with inflammatory cells,^118^ it has now also been proven that patients with rosacea have an altered composition of sebaceous fatty acids in the affected areas. As a result, the lipid barrier of the skin is disrupted.^16^ There is currently no data available on whether there are also alterations in the ERBB receptor system in this disease. However, it is known that the composition of sebum can be influenced by the regulation of ERBBs.^81^

## Conclusions and future directions

The ERBB receptor family plays a critical role not only in the development and homeostasis of epidermal and hair follicle keratinocytes,^119^^,^^120^ but also significantly influences the development and homeostasis of the SG. Progenitor cells of the SG express LRIG1, a potent negative feedback regulator of ERBB receptors that promotes receptor degradation, thereby inhibiting proliferation and enabling the differentiation of progenitor cells into mature sebocytes. Conversely, ligands of the EGFR appear to activate the receptor in progenitor cells, leading to increased proliferation and reduced sebocyte differentiation and maturation (Figure 1). Inhibition or loss of ERBB receptors, such as that therapeutically induced during cancer treatment, is associated with comedone formation in the SG. In contrast, excessive activation of EGFR can lead to SG hyperplasia, with the extent of this effect varying depending on the ligand involved. Thus, ERBB receptors appear to be directly responsible for SG cell proliferation and indirectly influence the differentiation and maturation of sebocytes, and it may be worth considering these receptors in future research on treatment strategies for diseases affecting the SG. Here, for example, topic treatment with a human recombinant EGF has already led to successes in acne therapy,^121^ but also in the treatment of EGFR inhibitor-related skin adverse events.^122^ Moreover, it is now known that proteins secreted by certain lactobacilli can stimulate the EGFR.^123^^,^^124^ This potential could be considered in the composition of topical probiotic lotions, which have already been successfully used in the treatment of acne, AD, or seborrheic dermatitis.^125^^,^^126^ However, as numerous skin cell types beyond sebocytes are responsive to ERBB signaling, therapeutically exploring this pathway is likely to result in skin side effects, making it essential to pinpoint sebaceous gland-specific mechanisms of ERBB action.

As the precise mechanisms underlying the role of ERBBs in sebaceous gland proliferation, differentiation, and maturation processes remain incompletely understood, further basic research is required. Here, the development of an appropriate SG-specific driver mouse line would be particularly important to enable even more specific *in vivo* studies. On the other hand, novel experimental models such as XL-i-20 sebocytes^127^ or 3D-SeboSkin^128^ could be of great benefit. The XL-i-20 model has been used to generate sebocyte organoids that provide a better representation of cell-cell interactions and further allow for better incorporation of apoptosis mechanisms into studies through immortalization with the hTERT system, while 3D-SeboSkin permits studying the crosstalk between *ex vivo* skin explants and sebocytes. Such emerging models offer a promising platform to further explore the therapeutic potential of targeting the ERBB receptor system in SG disorders and the pathological side effects of emerging cancer therapeutics.

## Acknowledgments

The authors have no acknowledgments to declare.

## Author contributions

Conceptualization: M.D., M.R.S., and T.H.; resources: M.D.; writing – original draft preparation: M.D., T.H., and M.R.S.

## Declaration of interests

The authors state no conflict of interest.
